# Mycobacteriophage–antibiotic therapy promotes enhanced clearance of drug-resistant *Mycobacterium abscessus*

**DOI:** 10.1242/dmm.049159

**Published:** 2021-09-17

**Authors:** Matt D. Johansen, Matthéo Alcaraz, Rebekah M. Dedrick, Françoise Roquet-Banères, Claire Hamela, Graham F. Hatfull, Laurent Kremer

**Affiliations:** 1 Institut de Recherche en Infectiologie de Montpellier, Centre National de la Recherche Scientifique UMR 9004, Université de Montpellier, Montpellier 34293, France; 2 Department of Biological Sciences, University of Pittsburgh, Pittsburgh, PA 15260, USA; 3 INSERM, Institut de Recherche en Infectiologie de Montpellier, Montpellier 34293, France

**Keywords:** *Mycobacterium abscessus*, Phage therapy, Cystic fibrosis, CFTR, Pathogenesis, Zebrafish

## Abstract

Infection by multidrug-resistant *Mycobacterium abscessus* is increasingly prevalent in cystic fibrosis (CF) patients, leaving clinicians with few therapeutic options. A compassionate study showed the clinical improvement of a CF patient with a disseminated *M. abscessus* (GD01) infection, following injection of a phage cocktail, including phage Muddy. Broadening the use of phage therapy in patients as a potential antibacterial alternative necessitates the development of biological models to improve the reliability and successful prediction of phage therapy in the clinic. Herein, we demonstrate that Muddy very efficiently lyses GD01 *in vitro*, an effect substantially increased with standard drugs. Remarkably, this cooperative activity was retained in an *M. abscessus* model of infection in CFTR-depleted zebrafish, associated with a striking increase in larval survival and reduction in pathological signs. The activity of Muddy was lost in macrophage-ablated larvae, suggesting that successful phage therapy relies on functional innate immunity. CFTR-depleted zebrafish represent a practical model to rapidly assess phage treatment efficacy against *M. abscessus* isolates, allowing the identification of drug combinations accompanying phage therapy and treatment prediction in patients.

This article has an associated First Person interview with the first author of the paper.

## Abstract (precis)

**Summary:** A zebrafish model of infection was developed to evaluate the *in vivo* cooperative activity of specific phages and antibiotics for the treatment of *Mycobacterium abscessus* infection.

## INTRODUCTION

The incidence of infection with non-tuberculous mycobacteria (NTM) is on the rise, frequently surpassing the infection rate of tuberculosis in many industrialised countries (Johansen et al., 2020a). The *Mycobacterium abscessus* complex is among the most-relevant NTM affecting humans, and is an acknowledged pathogen infecting a vast array of tissues associated with a broad spectrum of clinical manifestations. Members of this complex are rapid growers, often associated with severe pulmonary diseases, particularly in cystic fibrosis (CF) patients (Cowman et al., 2019; Johansen et al., 2020a). From a taxonomic perspective, the *M. abscessus* complex comprises three subspecies exhibiting different clinical outcomes and susceptibilities to antibiotic treatments: *M. abscessus* subsp. *abscessus* (designated hereafter *M. abscessus*), *M. abscessus* subsp. *bolletii* (designated hereafter *M. bolletii*) and *M. abscessus* subsp. *massiliense* (designated hereafter *M. massiliense*) (Adekambi et al., 2017). These complex subspecies are among the most drug-resistant mycobacteria, harbouring a vast array of innate and acquired drug resistance mechanisms against most antibiotic classes (Johansen et al., 2020a; Nessar et al., 2012). These mechanisms largely rely on the expression of diverse enzymes that inactivate drugs, such as β-lactams (Dubée et al., 2015), tetracyclines (Rudra et al., 2018), rifamycins (Rominski et al., 2017a) and aminoglycosides (Rominski et al., 2017b), and induce the expression of multiple MmpL-based drug efflux pumps (Gutiérrez et al., 2019; Richard et al., 2019).

Conventional therapeutic treatments often require prolonged courses of regimens combining a macrolide (clarithromycin or azithromycin), a β-lactam (cefoxitin or imipenem) and an aminoglycoside (amikacin) (Griffith et al., 2007). Macrolides remain the cornerstone of *M. abscessus* multidrug regimens (Griffith et al., 2007); however, treatment success rates are poor, particularly in macrolide-resistant strains involving an inducible ribosomal methylase encoded by *erm(41)* (Nash et al., 2009; Richard et al., 2020). As such, treatments against *M. abscessus* pulmonary diseases remain extremely challenging, often leading to severe adverse effects and therapeutic failure. New therapeutic interventions are thus required for the treatment and eradication of multidrug-resistant *M. abscessus* strains, particularly in chronically infected patients. In this context, phage therapy may represent a potential antibacterial alternative that involves the administration of phages that infect and lyse bacteria to eradicate or prevent infectious diseases. In the growing era of personalised medicine, phage therapies offer distinct advantages over broad-spectrum antibiotics as they are highly specific toward a particular bacterial pathogen without adversely affecting the host or host commensal microbiota, and ultimately prevent the emergence of antibiotic resistance (Luong et al., 2020). Supporting this view, genetically engineered mycobacteriophages were recently administered to a young CF patient chronically infected with a multidrug-resistant *M. massiliense* strain (GD01) following bilateral lung transplantation. Intravenous phage treatment was well tolerated and associated with significant clinical improvements, representing the first reported therapeutic use of phages for a human mycobacterial infection and the first use of engineered mycobacteriophages (Dedrick et al., 2019).

As phage-bacterial pairings are highly specific, there is substantial variation in *M. abscessus* phage susceptibilities, and, thus, phage therapy of large patient cohorts will require expansion of our understanding of mycobacteriophage interactions in the host. In these instances, the development of rapid and reliable pre-clinical models to examine the efficacy of phage therapy against a particular *M. abscessus* clinical isolate would represent a useful predictive marker of successful phage inclusion in a patient prior to clinical application. Moreover, although phage treatment alone is unlikely to be sufficient to clear the infection (Dedrick et al., 2019), an ideal pre-clinical model should allow the identification of top drug candidates that would accompany phage therapy, recapitulating phage–antibiotic cooperation that would facilitate successful eradication in a patient setting.

One of the key steps in drug discovery and phage therapy relies on the evaluation of the *in vitro* and *in vivo* potential of new treatments against *M. abscessus* using adapted animal models. Early studies confirmed that most immunocompetent mouse strains resulted in clearance of *M. abscessus* in the first weeks after infection (Bernut et al., 2014b; Obregón-Henao et al., 2015; Ordway et al., 2008), making these animal models very limited for studying pathogenesis or assaying the *in vivo* efficacy of drug treatments against *M. abscessus*. Comparatively, recent work demonstrated that C3HeB/FeJ mice allow the establishment of a chronic *M. abscessus* infection (Le Moigne et al., 2020). Non-mammalian models of infection have also been developed, including *Drosophila melanogaster*, *Galleria mellonella* larvae (Meir et al., 2018) or zebrafish (*Danio rerio*), offering advantages in terms of speed, cost, technical convenience and ethical acceptability over the mouse model (Bernut et al., 2017). In particular, zebrafish are increasingly recognised as a practical and amenable model to study host–pathogen interactions (Alibaud et al., 2011; Cambier et al., 2014; Clay et al., 2007; Davis et al., 2002; Gomes and Mostowy, 2020; Prajsnar et al., 2008, 2013; van der Sar et al., 2004; Vergunst et al., 2010). Owing to genetic tractability and optical transparency, zebrafish embryos represent an exquisite model to study important aspects of infectious diseases. Although adult zebrafish possess a complex immune system similar to that of humans, comprising both the innate and adaptive arms of immunity, the embryonic stages solely harbour innate immunity (Davis et al., 2002). Critical insights into the *M. abscessus* pathogenesis emerged from a zebrafish infection model, which identified the importance of cording as a mechanism of immune evasion (Bernut et al., 2014a) and the role of TNF signalling in controlling infection and granuloma formation (Bernut et al., 2016b). Zebrafish models of mycobacterial infection recapitulate important bacterial virulence mechanisms and host susceptibility determinants that have been further validated in humans (Bernut et al., 2016b), thus demonstrating their clinical relevance and translatability as an excellent model for human infections. Recently, we reported a CF transmembrane conductance regulator (CFTR)-depleted zebrafish model of *M. abscessus* infection that recapitulates CF immunopathogenesis (Bernut et al., 2019). Loss of CFTR increases susceptibility to *M. abscessus* through impaired NADPH oxidase-dependent restriction of intracellular growth, leading to uncontrolled extracellular multiplication of *M. abscessus* and resulting in abscess formation and lethal infection. Similar phenotypes were also observed in the zebrafish CF model of *Mycobacterium fortuitum* infection (Johansen and Kremer, 2020a), another frequently isolated NTM species that can be isolated from CF patients (Martiniano et al., 2016). Collectively, these studies highlight the importance of zebrafish infection models as excellent tools to decipher host–pathogen interactions and, for the first time, identified the pivotal role of CFTR in the immunological control of CF-associated NTM infections.

As such, we reasoned that the zebrafish CF model may represent a novel pre-clinical animal model to further understand the complex interplay between phages and bacteria in real time. Furthermore, this model may also provide an effective biological tool to assess the efficacy of mycobacteriophage activity *in vivo*. Herein, we evaluated the selectivity and efficacy of various mycobacteriophages against the *M. massiliense* clinical isolate GD01 *in vitro* and explored the improved activity with leading drug candidates. Importantly, we report the essential contribution of innate immunity in determining phage therapy success and the activity of phages and standard anti-mycobacterial drugs both *in vitro* and *in vivo.* Finally, in the current study, we demonstrate that the zebrafish CF model represents a valuable platform to decipher the lytic activity of mycobacteriophages *in vivo*, which epitomises phage therapy in a clinical setting.

## RESULTS

### High *in vitro* killing activity of phage Muddy against *M. massiliense* GD01

**Fig. 1.**
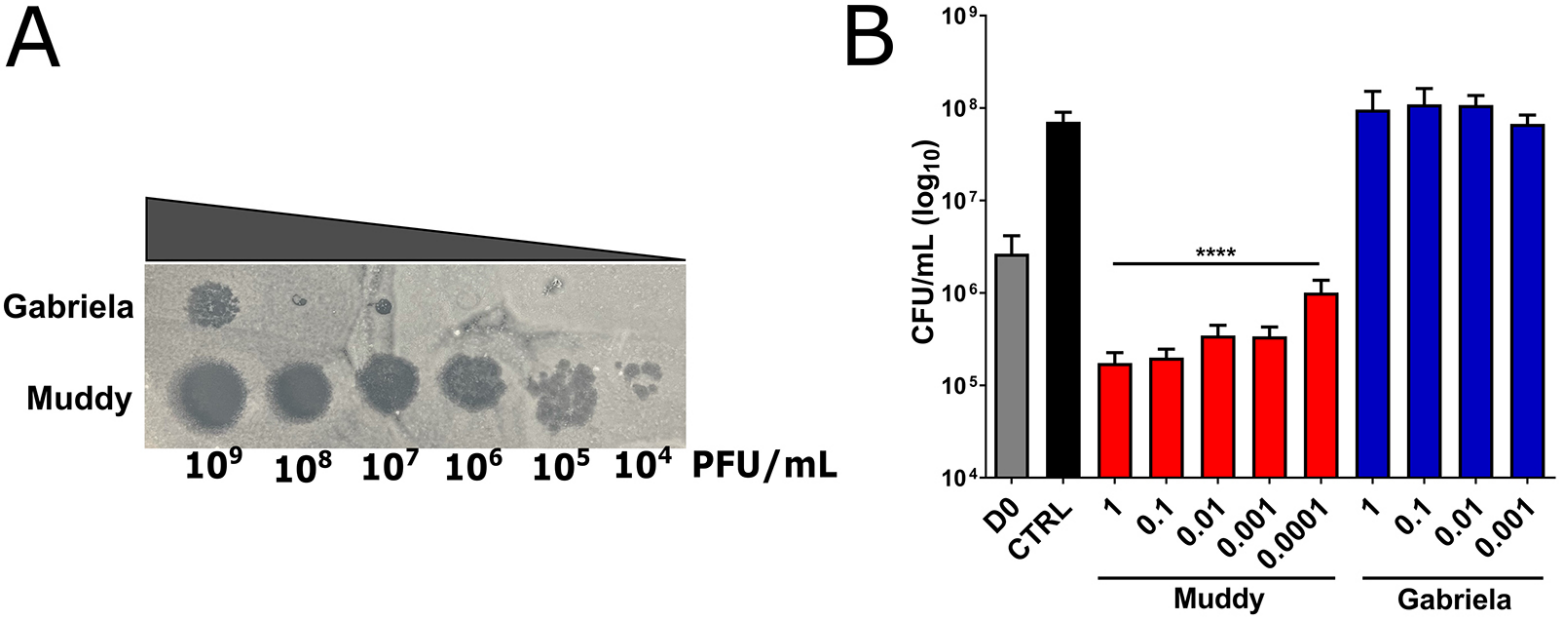
**Specificity and killing activity of mycobacterial phages against GD01.** (A) Plaque assay. Tenfold serial dilutions of phages Gabriela and Muddy were spotted on a lawn of GD01 grown on 7H10^OADC/CaCl2^. Lysis was observed after 5 days. PFU, plaque-forming units. (B) Killing assay. GD01 was grown in 7H9^OADC^, incubated with the various phages for 5 days at 37°C and plated on LB agar prior to colony-forming unit (CFU) counting. D0, GD01 inoculum; CTRL, GD01 without phages. ****P<0.0001 (unpaired Student's *t*-test). Data shown are the mean of three independent experiments±s.d.

The *M. abscessus* subsp. *massiliense* GD01 strain, with a rough colony morphology, isolated 1 month post-transplantation in a CF patient (homozygous for ΔF508), was originally used to select for potential therapeutic phages by exploiting a collection of >10,000 phages isolated using *Mycobacterium smegmatis*, thanks to the Science Education Alliance-Phage Hunters Advancing Genomics and Evolutionary Science (SEA-PHAGES) program (Jordan et al., 2014). This screen led to the selection of Muddy, which efficiently kills GD01, ZoeJ, from which a lytic derivative was engineered, as well as a lytic derivative of a host range mutant of the phage BPs, designated BPsΔ33HTH_HRM10, and used as a cocktail for treating the CF patient (Dedrick et al., 2019). An additional phage was identified, Gabriela, which infects and kills GD01 very inefficiently compared to Muddy (Fig. 1A) and provides a useful control phage.

We subsequently determined the lowest multiplicity of infection (MOI; corresponding to the phage:GD01 ratio) associated with the highest killing rate. Colony-forming units (CFU) were determined by counting the colonies after incubation of GD01 with either Muddy or Gabriela for 5 days in Middlebrook 7H9 at 37°C by varying the MOI range. As shown in Fig. 1B, even at the MOI of 0.0001, Muddy was able to reduce GD01 CFU numbers by ≈2 Log compared to the untreated control at Day 5. This lytic activity was further exacerbated at an MOI of 0.01 but remained stable at higher MOI (up to 1). In agreement with the plaque assay (Fig. 1A), Gabriela failed to show substantial killing activity, even at an MOI of 1, the highest MOI tested. Overall, these assays confirmed the differential specificity of the mycobacterial phages tested and high lytic activity of Muddy against GD01 *in vitro*.

### Improved activity between Muddy and antibiotics against GD01 *in vitro*

**Fig. 2.**
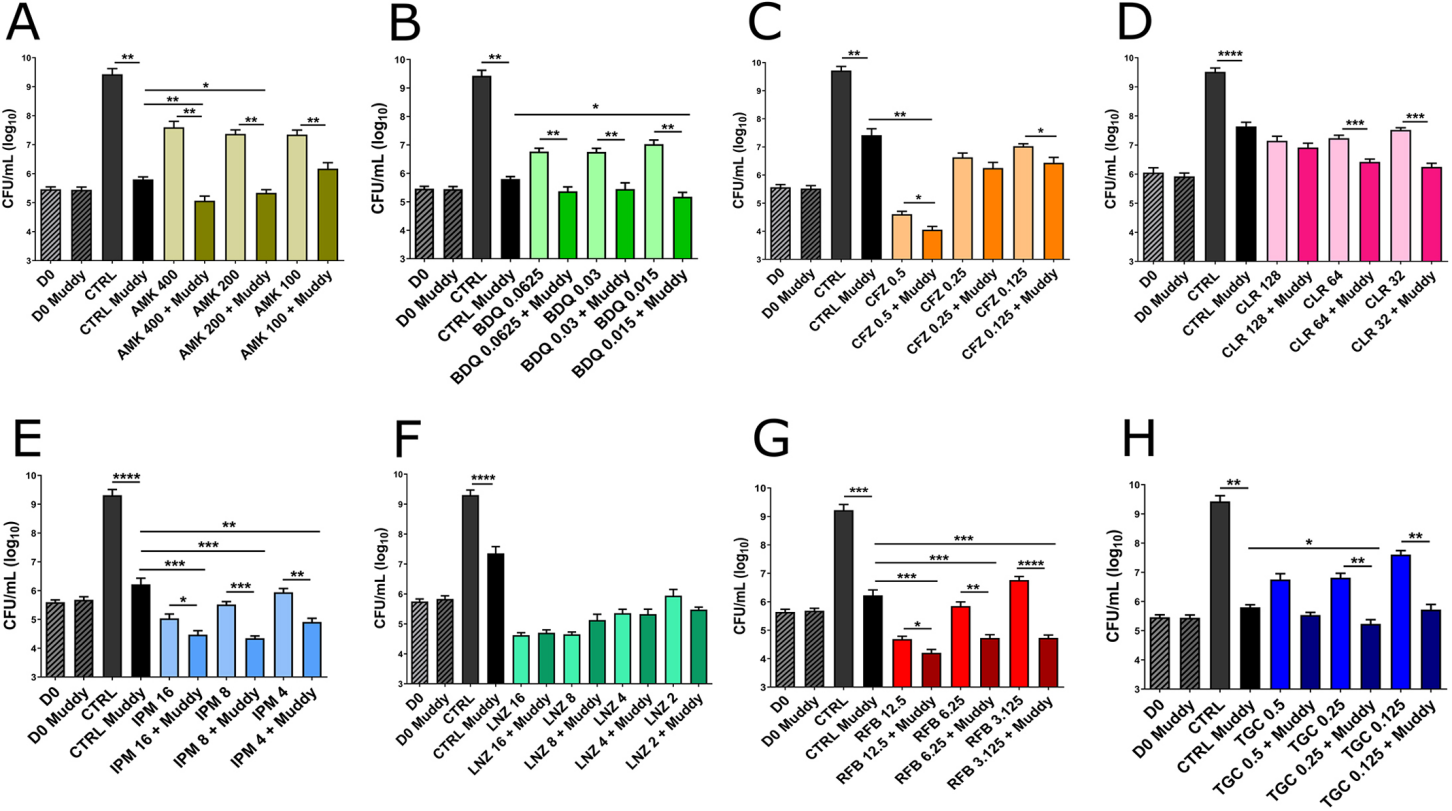
***In vitro* killing activity of Muddy and antibiotics against GD01.** (A-H) GD01 was grown in CaMHB at 37°C and incubated with Muddy (MOI=0.0001) in the absence or presence of different antibiotics used in clinical settings. Three drug concentrations were used (in µg/ml) at sub-MIC doses. After 5 days of treatment, bacteria were plated on LB agar prior to CFU counting. D0, GD01 inoculum; CTRL, GD01 without phages. *P<0.05; **P<0.01; ***P<0.001; ****P<0.0001 (unpaired Student's *t*-test). Data shown are the mean of three independent experiments±s.d.

The CF patient chronically infected with GD01 failed to respond to intensive antibiotic therapies, even after extensive exposure to multidrug chemotherapy (Dedrick et al., 2019). To understand this lack of responsiveness to standard antimicrobial chemotherapy, we determined the drug susceptibility profile of GD01 towards a wide panel of drugs and compared it to that of the *M. abscessus* 104536^T^ and *M. massiliense* CIP108297^T^ reference strains (both are rough variants). Based on the minimal inhibitory concentration (MIC) and the Clinical and Laboratory Standards Institute (CLSI) breakpoints (Woods et al., 2011), GD01 shows intermediate susceptibility to imipenem (IPM) and cefoxitin (CFX), and resistance to clarithromycin (CLR), amikacin (AMK), ciprofloxacin (CIP) and linezolid (LNZ) (Table S1). Tigecycline (TGC), clofazimine (CFZ), bedaquiline (BDQ) and MmpL3 inhibitors (the indole-2 carboxamide Cpd12 and the benzimidazole EJMCh-6) are active against GD01 at low concentrations, while rifabutin (RFB) shows a slightly lower activity against GD01 than against the reference strains. Because previous studies have emphasised the increased efficacy of combining phages and antibiotics against *Staphylococcus aureus* (Dickey and Perrot, 2019), we wanted to determine whether phages and antibiotics may cooperate to improve the activity against GD01 *in vitro*. Bacterial cultures were treated for 5 days with either Muddy alone (at an MOI of 0.0001), antibiotics alone (at sub-MIC) or with Muddy plus antibiotics (Fig. 2). As mentioned above, Muddy alone was associated with a nearly 2 to 3 Log decrease in the CFU. Interestingly, although antimicrobial agents such as RFB, IPM, BDQ, CFZ, TGC, AMK, CLR and LNZ were associated with decreased CFU numbers, the addition of Muddy further reduced the CFU levels for most drugs (RFB, IPM, BDQ, CFZ, TGC, AMK) but not for CLR (at 128 µg/ml) and LNZ. For instance, the combination of Muddy with RFB, a drug that has recently been shown to inhibit both extracellular and intracellular forms of *M. abscessus* (Aziz et al., 2017; Johansen et al., 2020b), at 3.1 µg/ml led to a ≈4-5 Log decrease in the CFU compared to the untreated control and a 2 Log reduction compared to RFB alone (Fig. 2G). Interestingly, although GD01 is resistant to AMK (MIC>128 µg/ml; Table S1), with limited CFU reduction, the addition of Muddy to AMK at sub-MIC was accompanied by a striking reduction in the CFU levels (Fig. 2A). Overall, these findings indicate that the addition of a wide panel of antimicrobial drugs potentiates the effect of Muddy and vice versa*.* In addition, drug/phage combinations can overcome the limited activity of drugs in antibiotic-resistant isolates.

### Muddy reduces the pathological signs of GD01 infection in wild-type zebrafish

We have previously exploited the optical transparency of zebrafish embryos to describe the increased virulence of rough (R) members of the *M. abscessus* complex over their smooth (S) counterparts, which correlated with the loss of glycopeptidolipid (GPL) production (Bernut et al., 2014a, 2016a). Herein, we exploited this model to assess whether Muddy exhibits killing activity against GD01 *in vivo* and whether it alters the pathological signs associated with GD01 infection in wild-type embryos. We first addressed whether GD01 induces a typical infection following caudal vein injection with 250-300 CFU of tdTomato-expressing GD01 at 30 h post-fertilisation (hpf). Virulence of GD01 in zebrafish was compared to the virulence of the S and R variants of the *M. massiliense* CIP108297^T^ strain as well as T56, an unrelated rough *M. abscessus* clinical isolate. As observed previously for *M. abscessus* and *M. bolletii* (Bernut et al., 2014a, 2016a), daily monitoring of mortality indicated that embryos injected with the S CIP108297^T^ survived up until 12 days post-infection (dpi) (Fig. S1A), whereas ∼50% of embryos injected with the R CIP108297^T^ succumbed to infection by 10 dpi. Infection with T56 showed increased virulence, with nearly 80% embryo mortality at 12 dpi. In contrast, GD01 exhibited a significant delay in embryo killing compared to the other R strains. GD01-infected zebrafish started to succumb to infection 5 days after T56-infected zebrafish, with only 40% embryo mortality by 12 dpi. A possible explanation for this delay may be the slower growth rate of GD01 compared to that of the other strains *in vitro.* This was confirmed by monitoring the growth kinetics of GD01 at both 30°C and 37°C by CFU determination, despite important variations caused by the highly aggregative and clumping properties that typify rough strains (Fig. S2). However, all R strains developed a systemic infection in fish with the presence of the typical growing foci of infection in the larval brain, as shown by whole-embryo imaging (Fig. S1B).

**Fig. 3.**
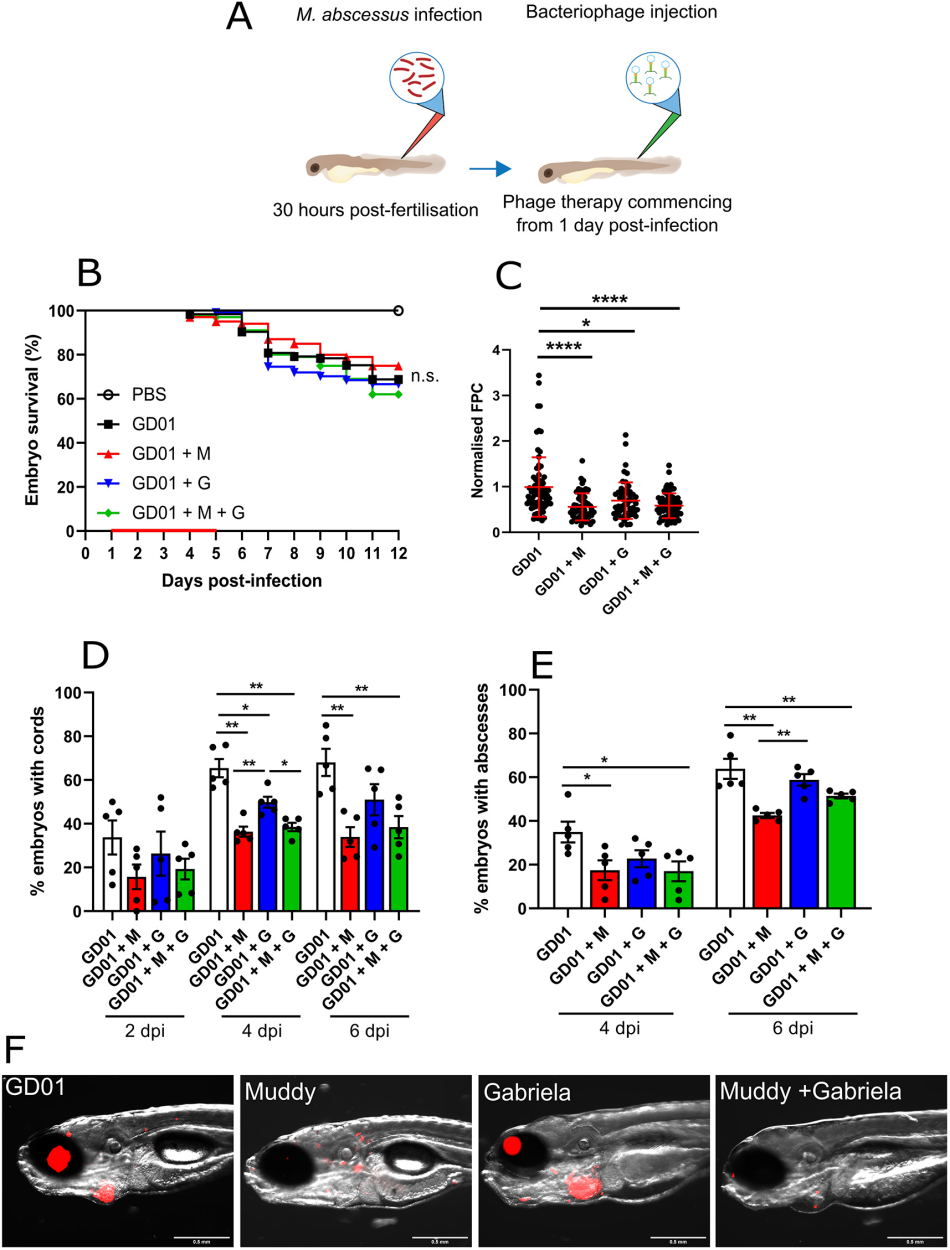
**Impact of bacteriophage Muddy on GD01 infection in wild-type zebrafish.** At 30 hpf, embryos were infected with 250-300 CFU of GD01 expressing tdTomato via caudal vein injection. At 1 dpi, embryos commenced phage therapy through caudal vein administration at an MOI of 50:1 (phage:bacteria) based on the initial bacterial infection inoculum. Embryos were treated daily from 1 dpi up to and including 5 dpi with either Muddy (M), Gabriela (G) or a phage cocktail containing both Muddy and Gabriela (M+G). (A) A generalised schematic showing the experimental design corresponding to the figure. (B) Embryo survival was monitored over a 12 day period, with embryos counted daily. Survival curves were analysed using the log-rank (Mantel–Cox) statistical test. The red bar across the *x*-axis indicates the duration of daily phage administration in the current experiment. (C) Bacterial burden [fluorescent pixel count (FPC)] was analysed at 6 dpi using fluorescent microscopy. Fluorescent images were analysed in ImageJ using the ‘Analyze particles’ function. Bacterial burden was analysed using a Kruskal–Wallis one-way ANOVA. (D,E) The proportion of embryos with cords (D) and abscesses (E) was enumerated at 2, 4 and 6 dpi using fluorescent microscopy. Abscess and cord quantification was analysed using a Kruskal–Wallis one-way ANOVA. Data shown are the mean of three independent experiments±s.d. (*n*=20-30 per group for each experiment). (F) Representative zebrafish images of untreated (GD01) or phage-treated embryos at 6 dpi, showing the presence of extracellular bacterial cords. Red overlay represents GD01 expressing TdTomato. Scale bars: 0.5 mm. n.s., not significant; *P<0.05; **P<0.01; ****P<0.0001.

Having established the GD01 infection model in zebrafish, we next tested whether intravenous injection of Muddy or the control phage Gabriela, which infects GD01 inefficiently, would affect the survival and pathology of the infection as outlined in Fig. 3A. We first investigated whether phage treatment increases the survival of GD01-infected larvae. No significant differences in larval survival were observed in the different phage-treated animals compared to the untreated animals (Fig. 3B). We next examined whether phage treatment exerts an effect on the bacterial burden by determining the fluorescent pixel count (FPC) at 6 dpi (Bernut et al., 2015). Fig. 3C clearly shows a significant reduction in the FPC after treatment with Muddy, whereas this effect was less pronounced with Gabriela, thus correlating with the *in vitro* lytic activity of the two phages against GD01. There was no further reduction in bacterial burden when both Muddy and Gabriela were combined together, suggesting that these phages do not act synergistically *in vivo*.

A noticeable feature of R strains of the *M. abscessus* complex is their high propensity to produce extracellular cords (Bernut et al., 2014a). Owing to their extensive size, cords prevent the bacilli from being phagocytosed by macrophages and neutrophils, thus representing an important mechanism of immune evasion (Bernut et al., 2014a). Therefore, cord formation, often representative of acute infection within the zebrafish embryo, was monitored at different time points after infection. Interestingly, treatment with Muddy was associated with a significant reduction (∼2-fold) in the proportion of embryos with cords at 2, 4 and 6 dpi (Fig. 3D). However, no significant changes were observed after treatment with Gabriela alone at 6 dpi, while injection of a cocktail containing both Muddy and Gabriela did not further reduce cording compared to Muddy alone.

Abscess formation often represents loss of infection control and typically occurs following extracellular cord formation and expansion (Bernut et al., 2014a, 2015, 2016a). Abscesses represent a marker of disease severity and are often associated with cellular debris, tissue destruction and acute infection in zebrafish (Bernut et al., 2014a). As such, we wanted to determine whether phage treatment would affect the production of abscesses. As shown in Fig. 3E, treatment with Muddy alone and a phage cocktail of both Muddy and Gabriela showed a significant decrease in the percentage of larvae with abscesses at 4 and 6 dpi. As expected, this was not the case for Gabriela-treated embryos, in which we observed no difference in abscess formation at any time points examined. The decrease in the pathological signs of Muddy-treated larvae correlates with the FPC analysis and whole-embryo imaging (Fig. 3F).

Together, these results suggest that Muddy very efficiently lyses GD01 in wild-type zebrafish embryos, translating into a decreased pathology of the infection. However, this was not sufficient to reduce larval killing. A possible explanation is that, given the delay in larval killing by GD01 compared to other R strains (Fig. S1), a clear effect of phage treatment on larval survival cannot be seen in the 12 dpi-restricted time frame of observation. Moreover, this is further exemplified by the short 5 day phage treatment duration in the zebrafish model, which we postulate may show further reductions in bacterial burden and translationally increased survival.

### Functional innate immunity is essential for efficient activity of Muddy in zebrafish

**Fig. 4.**
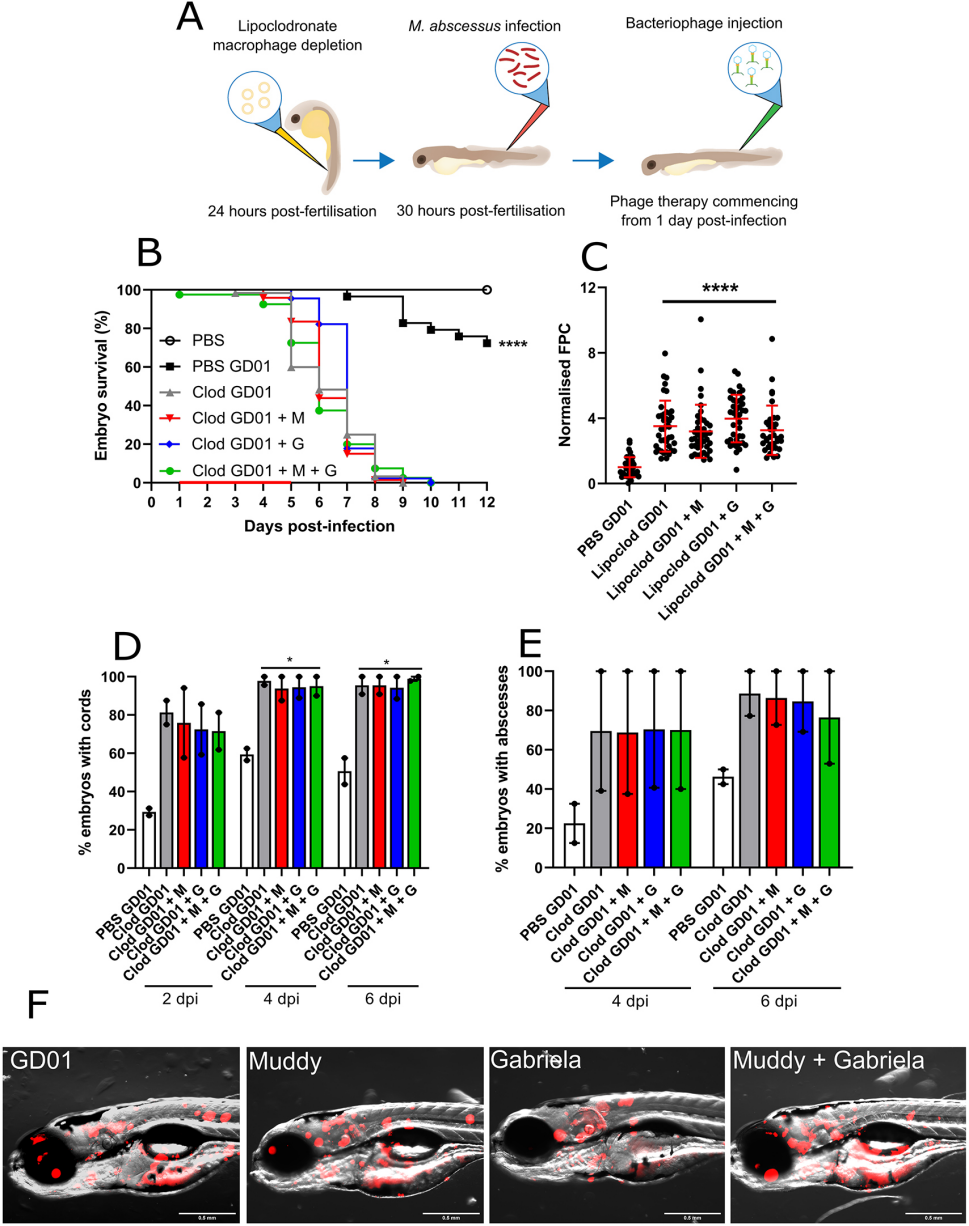
**Activity of Muddy in GD01-infected zebrafish lacking macrophages.** At 24 hpf, embryos were micro-injected with either PBS liposomes (PBS) or lipoclodronate-filled liposomes (Clod) to achieve macrophage depletion. At 30 hpf, treated embryos were infected with 250-300 CFU of GD01 (PBS GD01 and Clod GD01) via caudal vein injection. At 1 dpi, embryos commenced phage therapy through caudal vein injection at an MOI of 50:1 (phage:bacteria) based on initial bacterial infection inoculum. Embryos were treated daily from 1 dpi, up to and including 5 dpi with Muddy (M), Gabriela (G) or a phage cocktail containing both Muddy and Gabriela (M+G). (A) A generalised schematic showing the experimental design corresponding to the figure. (B) Embryo survival was monitored over a 12 day period, with embryos counted daily. Survival curves were analysed using the log-rank (Mantel–Cox) statistical test. The red bar across the *x*-axis indicates the duration of daily phage administration in the current experiment. (C) Bacterial burden (FPC) was analysed at 6 dpi using fluorescent microscopy. Fluorescent images were analysed in ImageJ using the ‘Analyze particles’ function. Bacterial burden was analysed using a Kruskal–Wallis one-way ANOVA. (D,E) The proportion of embryos with cords (D) and abscesses (E) was enumerated at 2, 4 and 6 dpi using fluorescent microscopy. Abscess and cord quantification was analysed using a Kruskal–Wallis one-way ANOVA. Data shown are the mean of two independent experiments±s.d. (*n*=20-30 per group for each experiment). (F) Representative zebrafish images of untreated (GD01) or phage-treated embryos at 6 dpi, showing the presence of extracellular bacterial cords. Red overlay represents GD01 expressing TdTomato. Scale bars: 0.5 mm. *P<0.05; ****P<0.0001.

Macrophages are important immune cells recruited to an infection and are the predominant cell subset involved in the control of *M. abscessus* infection in zebrafish (Bernut et al., 2014a). Importantly, macrophages are required for granuloma formation and controlling mycobacterial growth (Pagán and Ramakrishnan, 2018). Previous studies demonstrated that macrophage ablation results in rapid larval death within several days of infection, underpinning the crucial role of these phagocytes in containing mycobacterial infections (Bernut et al., 2014a; Clay et al., 2007; Johansen and Kremer, 2020b). We observed that injection of liposomal clodronate in the caudal vein had no effect on embryo survival (Fig. S3A) and depleted macrophages for at least 5 days, with macrophages recovering at 7 days post-treatment (Fig. S3B). Following liposomal clodronate treatment and *M. abscessus* infection (Fig. 4A), we observed progressive rapid larval death resulting in 100% of embryo mortality at 8 dpi, compared to standard GD01 infection following liposomal PBS injection prior to infection (Fig. 4B). Unexpectedly, although larval death occurred rapidly and was much more pronounced compared to infection in wild-type fish, treatment with Muddy, Gabriela or Muddy plus Gabriela failed to increase the survival rate of infected embryos (median time to 50% mortality of 6 and 7 days, respectively). We further examined whether there was a change in bacterial burden in macrophage-depleted embryos following phage therapy. Irrespective of individual phages or phage cocktail administration, we saw no change in bacterial burden between any of the macrophage-depleted groups at 6 dpi (Fig. 4C). As anticipated, both cords and abscesses increased drastically within 2 dpi in macrophage-depleted larvae, compared to the control embryos (liposomal PBS) (Fig. 4D,E). However, phage treatment did not reduce the proportion of embryos with cords at 2, 4 and 6 dpi (Fig. 4D), or the percentage of embryos with abscesses at 4 and 6 dpi (Fig. 4E). The presence of large infection foci in the phage-treated fish compared to the untreated fish correlates with the FPC analysis and with whole-embryo imaging (Fig. 4F). Collectively, these results highlight the critical role of macrophages in sustaining the *in vivo* efficacy of Muddy during GD01 infection and emphasise the requirements of a functional innate immunity for establishing a successful phage therapy.

### Validation of phage treatment against GD01 in CFTR-depleted embryos

**Fig. 5.**
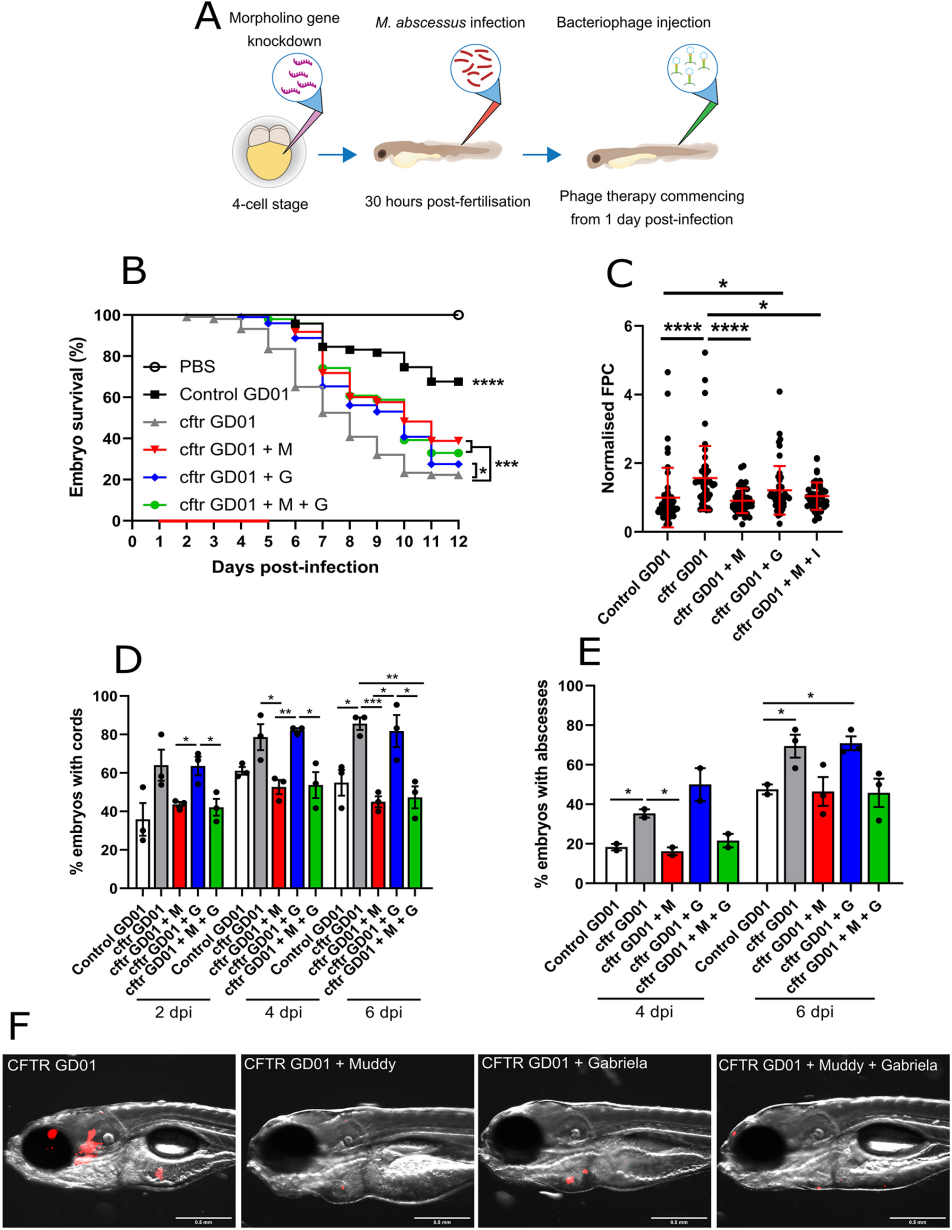
**Effect of Muddy in GD01-infected *cftr* morphants.** At the one- to four-cell stage, fertilised zebrafish eggs were injected in the nucleus with either control morpholino (control) or *cftr* morpholino (cftr). At 30 hpf, embryos were infected with 250-300 CFU of GD01 (control GD01 or cftr GD01) via caudal vein injection. At 1 dpi, embryos commenced phage therapy through caudal vein injection at an MOI of 50:1 (phage:bacteria) based on initial bacterial infection inoculum. Embryos were treated daily from 1 dpi, up to and including 5 dpi with Muddy (M), Gabriela (G) or a phage cocktail containing both Muddy and Gabriela (M+G). (A) A generalised schematic showing the experimental design corresponding to the figure. (B) Embryo survival was monitored over a 12 day period, with embryos counted daily. Survival curves were analysed using the log-rank (Mantel–Cox) statistical test. The red bar across the *x*-axis indicates the duration of daily phage administration in the current experiment. (C) Bacterial burden (FPC) was analysed at 6 dpi using fluorescent microscopy. Fluorescent images were analysed in ImageJ using the ‘Analyze particles’ function. Bacterial burden was analysed using a Kruskal–Wallis one-way ANOVA. (D,E) The proportion of embryos with cords (D) and abscesses (E) was enumerated at 2, 4 and 6 dpi using fluorescent microscopy. Abscess and cord quantification was analysed using a Kruskal–Wallis one-way ANOVA. Data shown are the mean of three independent experiments±s.d. (*n*=20-30 per group for each experiment). (F) Representative *cftr* morphant images of untreated (GD01) or phage-treated embryos at 6 dpi, showing the presence of extracellular bacterial cords or localised infection. Red overlay represents GD01 expressing TdTomato. Scale bars: 0.5 mm. *P<0.05; **P<0.01; ***P<0.001; ****P<0.0001.

The *M. abscessus* complex has emerged as an important respiratory pathogen of major concern in CF centres worldwide (Parkins and Floto, 2015). Because our understanding of the particular vulnerability of CF patients to *M. abscessus* complex infection remains limited by the lack of suitable animal models mimicking the immune abnormalities found in the CF population, we have exploited a zebrafish CF model that recapitulates important aspects of CF immunopathogenesis. This allowed us to report a stepwise dissection of *M. abscessus* infection in an animal depleted of CFTR to elucidate the biological implication of CFTR in innate immunity to these infections (Bernut et al., 2019). To address the role of CFTR in GD01 infection, *cftr* loss-of-function experiments were performed in zebrafish using a specific morpholino-modified oligonucleotide (Bernut et al., 2019), which abrogated production of native spliced *cftr* transcripts (Fig. 5A; Fig. S4). We report here that GD01*-*infected CFTR morphants rapidly succumb to intravenous infection (Fig. 5B), reflecting the hypersusceptibility of the young CF patient to this isolate (Dedrick et al., 2019), providing a first glimpse into CFTR-mediated host defences to GD01 infection. This was associated with an increase in the bacterial burden, as evidenced by FPC determination (Fig. 5C). This early and progressive killing in *cftr* morphants (Fig. 5B) prompted us to investigate whether phage therapy can ameliorate GD01 infection in a CFTR-deficient context.

Treatment with Muddy, Gabriela or Muddy and Gabriela was associated with a slightly delayed survival curve compared to CFTR-depleted embryos infected with GD01 (median time to 50% mortality of 10 days compared to 8 days, respectively) (Fig. 5B). Importantly, although CFTR ablation led to 20% embryo survival in the non-treated group at 12 dpi, ∼40% survival was reached in embryos treated with Muddy. Similar to macrophage-depleted embryos, ablation of CFTR was associated with a rapid and pronounced increase in cording (Fig. 5D) and abscesses (Fig. 5E) compared to control morphants. Strikingly, treatment with Muddy alone or in combination with Gabriela led to a severe reduction in the proportion of embryos with cords at 2, 4 and 6 dpi (Fig. 5D), abscesses at 4 and 6 dpi (Fig. 5E) and bacterial loads (Fig. 5C), compared to the untreated controls. The strong decrease in the pathological signs of Muddy-treated *cftr* morphants correlates with the FPC analysis and whole-embryo imaging (Fig. 5F). As evidenced by increased larval survival and reduced pathological signs, these findings indicate that CFTR-depleted fish are highly susceptible to GD01 infection and respond to phage treatment with Muddy.

### Improved efficacy of combined phage and antibiotic therapy in GD01-infected CFTR zebrafish

**Fig. 6.**
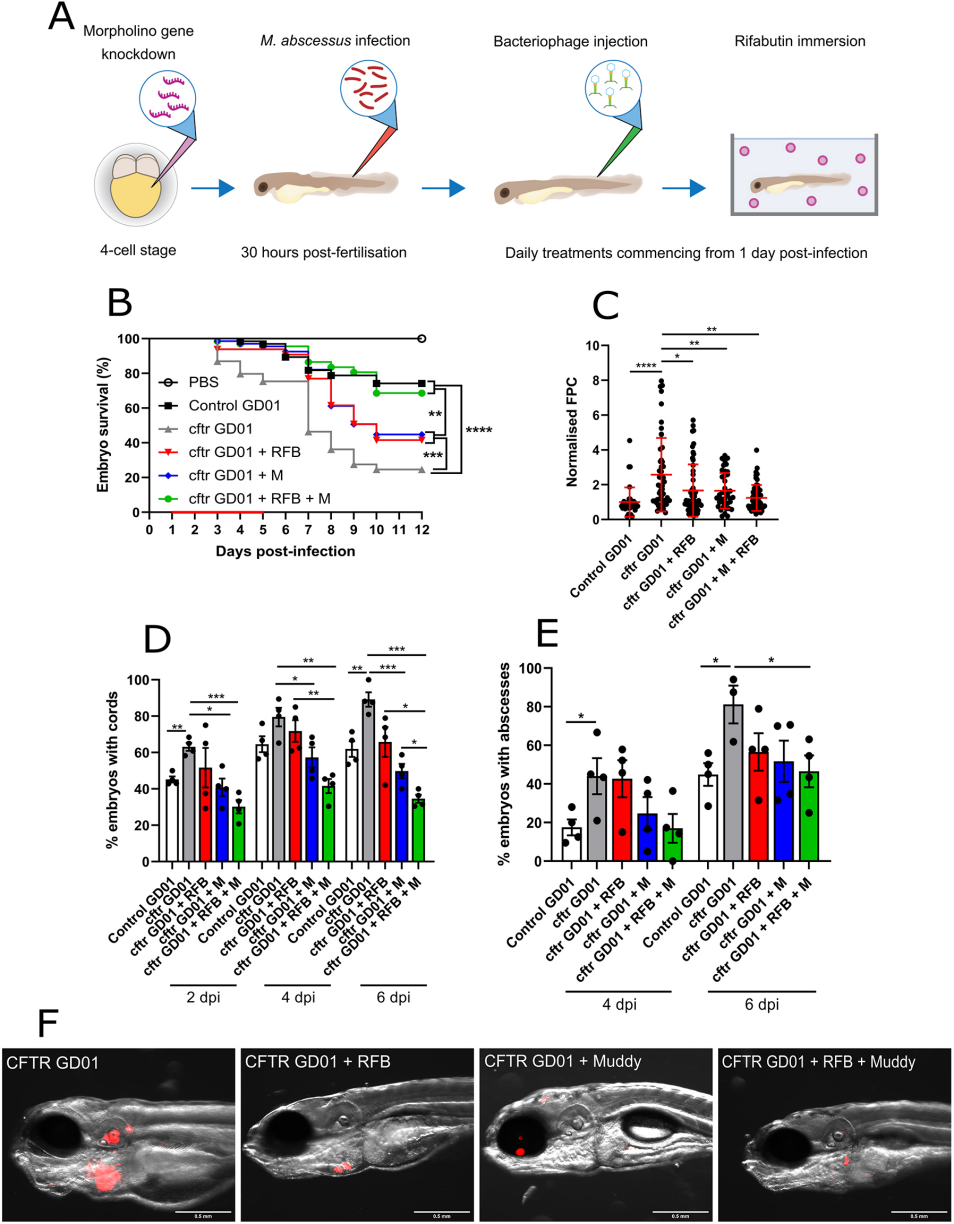
**Activity of Muddy and rifabutin in GD01-infected *cftr* morphants.** At the one- to four-cell stage, fertilised zebrafish eggs were injected in the nucleus with either control morpholino (control) or *cftr* morpholino via caudal vein injection. At 1 dpi, embryos commenced phage Muddy (M) and/or rifabutin (RFB) therapy through caudal vein injection at an MOI of 50:1 (phage:bacteria) based on initial bacterial infection inoculum. Embryos were treated daily from 1 dpi, up to and including 5 dpi with Muddy. RFB was added to zebrafish water daily at a final concentration of 50 µg/ml. (A) A generalised schematic showing the experimental design corresponding to the figure. (B) Embryo survival was monitored over a 12 day period, with embryos counted daily. Survival curves were analysed using the log-rank (Mantel–Cox) statistical test. The red bar across the *x*-axis indicates the duration of daily phage and RFB administration in the current experiment. (C) Bacterial burden (FPC) was analysed at 6 dpi using fluorescent microscopy. Fluorescent images were analysed in ImageJ using the ‘Analyze particles’ function. Bacterial burden was analysed using a Kruskal–Wallis one-way ANOVA. (D,E) The proportion of embryos with cords (D) and abscesses (E) was enumerated at 2, 4 and 6 dpi using fluorescent microscopy. Abscess and cord quantification was analysed using a Kruskal–Wallis one-way ANOVA. Data shown are the mean of three independent experiments±s.d. (*n*=20-30 per group for each experiment). (F) Representative *cftr* morphant images of embryos left untreated (GD01), treated with Muddy alone, RFB alone or with Muddy plus RFB at 6 dpi, showing the presence of extracellular bacterial cords or localised infection. Red overlay represents GD01 expressing TdTomato. *P<0.05; **P<0.01; ***P<0.001; ****P<0.0001.

Our previous results highlighted the improved activity when combining Muddy and various antibiotics, including RFB (Fig. 2). To enquire whether this therapeutic combination is also effective *in vivo*, GD01-infected CFTR-depleted zebrafish were either treated with RFB alone, Muddy alone or with RFB and Muddy together. The protocol applied is outlined in Fig. 6A. Treatment with RFB alone was associated with a significant increase in the survival rate compared to that of the untreated CFTR morphants (Fig. 6B), in agreement with previous observations performed in wild-type zebrafish infected with *M. abscessus* (Johansen et al., 2020b). In addition, the level of protection conferred by RFB was similar to that of embryos treated with Muddy alone. Importantly, the combined Muddy and RFB treatment further increased the survival rate compared to that of larvae treated with RFB alone or Muddy alone, reaching 70% survival at 12 dpi, almost identical to that seen for GD01 infection in wild-type zebrafish (Fig. 6B). Strikingly, when we compared the median time to 50% mortality between the groups, we observed a median time of 7 days for CFTR embryos infected with GD01, which shifted to 10 days for those treated with RFB or Muddy alone. Importantly, this was beyond the calculation threshold for those embryos treated with RFB and Muddy together, owing to significantly reduced mortality, further reinforcing the relevance of our findings. Similarly, this combination therapy showed markedly reduced bacterial loads compared to untreated *cftr* morphants and showed no differences compared to untreated control morphants (Fig. 6C). Furthermore, combination therapy with Muddy and RFB was also correlated with a substantial reduction in the proportion of embryos with cording at 2, 4 and 6 dpi (Fig. 6D), as well as in the percentage of embryos with abscesses (Fig. 6E). The strong decrease in the pathological signs of Muddy plus RFB-treated *cftr* morphants correlates with the FPC analysis and whole-embryo imaging (Fig. 6F). Together, these findings demonstrate the improved therapeutic efficacy of the combined treatment with Muddy and RFB in a CF animal model with disseminated GD01 infection, characterised by a highly reduced lethality and pathological signs in comparison to embryos treated with either one of the two treatments.

## DISCUSSION

Bacterial pathogens are frequently associated with lung complications and disease progression in CF (Elborn, 2016). These bacteria increasingly show resistance to antibiotics, requiring novel management approaches. Bacteriophage therapy, in which lytic phages are administered to kill target bacterial pathogens, represents one of these strategies (Düzgüneş et al., 2021; Luong et al., 2020). Case reports of phage therapy to treat drug-resistant pulmonary infections in CF have received significant attention in recent years (Chan et al., 2021; Dedrick et al., 2019; Ng et al., 2021). The use of a phage cocktail administered with antibiotics was reported for the treatment of disseminating *M. massiliense* GD01 in a 15-year-old individual with CF who was a post-lung transplant recipient (Dedrick et al., 2019). In this case, the therapy resulted in decreased size of skin lesions and improvement in lung function, liver function, chest imaging and weight gain. Although this illustrates that phage therapy against NTM infections holds vast potential, there is little understanding of phage–host dynamics in disease environments and a clear lack of pre-clinical studies concerning phage therapy of *M. abscessus* infections (Senhaji-Kacha et al., 2021). In this context, we studied the GD01–mycobacteriophage infection dynamics under various physiological and disease-mimicking conditions using a CF zebrafish model of infection.

In general, most findings identified in this biological model paralleled those reported previously in the treated CF patient (Dedrick et al., 2019). First, we found that *cftr* morphants were extremely susceptible (decreased larval survival) to GD01 infection (Fig. 5B), resulting from uncontrolled bacterial multiplication (Fig. 5C) and dissemination, and exacerbation of the pathological features (Fig. 5D,E), as shown previously for *M. abscessus* (Bernut et al., 2019) and *M. fortuitum* (Johansen and Kremer, 2020a). Second, we show the improved activity of phage Muddy (Fig. 2) with a wide panel of antibiotics used in the clinical treatment of *M. abscessus* pulmonary diseases. Although the drugs tested were more or less effective at low concentrations, the addition of Muddy led to a substantial improvement in efficacy, similarly to what has previously been observed when drugs were administered to phages against *S. aureus* or *Pseudomonas aeruginosa* (Dickey and Perrot, 2019; Oechslin et al., 2017). In addition, the adjunct of an antibiotic to phage treatment has also proven effective in reducing the outgrowth of antibiotic- and phage-resistant strains during treatment (Dickey and Perrot, 2019), and this combination could also better help to manage antibiotic-resistant bacterial biofilms. A major drawback of a phage/antibiotic combination is that more complex studies are required to establish effective co-dosing regimens relying on compatible pharmacokinetic and pharmacodynamics properties between the antibiotic and phages. However, the mechanisms underlying potential phage–antibiotic interactions remain largely unknown. Thus, the present study represents a first glimpse into the phage/antibiotic effect in a zebrafish CF model in the context of infection. Indeed, we provide evidence of the improved activity of Muddy when combined with RFB in this animal model (Fig. 6), thus replicating the outcomes observed *in vitro* (Fig. 2) while also reiterating clinical observations associated with phage and antibiotic combination therapies. Whereas Muddy alone was shown to reduce the number of cords and abscesses (Fig. 3D,E and Fig. 5D,E), this effect was further exacerbated when adding RFB to Muddy (Fig. 6D,E). Because mycobacteriophages have limited access to the intracellular compartment of macrophages (Broxmeyer et al., 2002), they are thought to mainly target extracellular bacilli, which, in the case of rough *M. abscessus*, can grow in the form of large serpentine cords (Bernut et al., 2014a). As such, this is likely to explain the decrease in the proportion of embryos with cords and the subsequent proportion of embryos with abscesses. However, it is still not known whether phages have the capacity to lyse the bacilli present on the periphery of the cords, ultimately reducing the size and eventually eradicating cords, or whether they prevent the initiation of cord formation by targeting individual bacilli. Importantly, the proportion of embryos with cords in *cftr* morphants treated with Muddy remained constant at 2, 4 and 6 dpi, suggesting that Muddy does not degrade or modify the bacterial cord structure, but rather prevents the formation of new cords (Fig. 5D).

We previously reported that exposure of *M. abscessus*-infected THP-1 macrophages to RFB was associated with reduced intra- and extracellular cording (Johansen et al., 2020b). Therefore, the net effect of the Muddy plus RFB therapy is likely to result in a pronounced decrease in both intra- and extracellular cording in zebrafish. Moreover, this significant reduction in cording is particularly interesting, as it may prevent the subsequent formation of abscesses (Bernut et al., 2014a), considered a marker of the severity of the disease. Consistent with this hypothesis, a marked decrease in abscess formation was observed in Muddy-, RFB- and Muddy plus RFB-treated zebrafish embryos (Fig. 6E). Validation of Muddy plus RFB co-treatment against GD01 infection in CFTR-depleted embryos was not only reflected by a strong decrease in the pathophysiological symptoms (abscesses and cords; Fig. 6D,E) but also by a strong reduction in larval mortality (Fig. 6B). Together, the positive outcome of phage plus antibiotic treatment in the infected CF zebrafish reflects those observations identified following treatment of the GD01-infected CF patient, emphasising the usefulness of the zebrafish model as an effective and predictive pre-clinical model. This is particularly justified in the context of an increasing demand for assessing the use of bacteriophages in CF and non-CF patients infected with multidrug-resistant bacteria, including *M. abscessus* (Aslam et al., 2020).

Numerous animal models have recently been developed for the evaluation of phage therapy for infections caused by the ESKAPE group of pathogens (consisting of notable drug-resistant pathogens, such as *Enterococcus faecium*, *S. aureus*, *Klebsiella pneumoniae*, *Acinetobacter baumannii*, *P. aeruginosa* and *Enterobacter* spp.) (Cieślik et al., 2021). These include *Galleria mellonella*, *Drosophila melanogaster* and various mouse models of sepsis/peritonitis/pneumonia/urinary tract infection or eye infection. In addition, *cftr* zebrafish morphants were used to demonstrate the applicability of phage therapy against *P. aeruginosa* infections (Cafora et al., 2019). However, in this study, phages were injected once in the yolk sac, which does not correspond to a route of administration in humans. In our study, Muddy was administered intravenously several times on a daily basis, similarly to the treatment conditions encountered in the case in the CF patient (Dedrick et al., 2019). It is important to emphasise that, in our study, phages were administered daily to recapitulate clinical implementation; however, we were only able to do this up to 5 days post-infection due to increasing epithelial thickening throughout embryonic development. It is tempting to speculate that we may see further reductions in bacterial burden, cording and abscess formation associated with increased embryo survival if we were able to administer phages for a longer period of time; however, this remains to be further explored.

Noteworthily, despite their unique features for high-throughput therapeutic evaluation, zebrafish embryos display disadvantages over mammalians predominantly stemming from stark anatomical differences (gills instead of lungs, haematopoiesis occurring in the anterior kidney instead of the bone marrow, and lack of discernible lymph nodes). In addition, the lack of adaptive immunity early in embryonic development may also influence the outcome of the phage/antibiotic treatment in our study. As such, these factors should be taken into consideration when interpreting the findings from the current study and for determining the suitability of different animal models to examine the efficacy of phage therapy. Although devoid of lungs, which are the main target organs for *M. abscessus* pulmonary disease, zebrafish has recently emerged as a powerful model system to better understand CF as well as the vulnerability of CF patients to *M. abscessus* infection (Bernut et al., 2019), and for assessing the *in vivo* efficacy of multiple drugs against *M. abscessus* (Bernut et al., 2014b; Dubée et al., 2015; Dupont et al., 2016, 2017; Johansen et al., 2020b; Raynaud et al., 2020). From a fundamental perspective, use of this model allowed us here to assess the contribution of innate immunity in phage therapy. Our results indicate that functional innate immunity is required for efficient activity of Muddy in zebrafish. Using macrophage-depleted embryos, we found that macrophages are critical for successful phage-mediated bacterial clearance. At this stage, we do not know whether the failure of phage therapy was due to the lack of macrophage effector functions such as phagocytosis or the absence of macrophage-dependent activation of other immune effector components participating in GD01 clearance. These findings are in agreement with a previous investigation devoted to studying the effects of host immunity on the efficacy of phage therapy for acute pneumonia caused by multidrug-resistant *P. aeruginosa* using various immune-compromised mouse models, which revealed that neutrophils are required for phages to clear infection (Roach et al., 2017). It is important to recognise that macrophage ablation is known to result in rapid embryo death and significantly enhances susceptibility to mycobacterial infection due to the absence of a major immunological defence (Bernut et al., 2014a). Critically, macrophage ablation led to ineffective phage therapy, suggesting that macrophages may be required to facilitate mycobacterial clearance in the presence of mycobacteriophages. Alternatively, it is also plausible that the phage dose administered to lipoclodronate-treated embryos could be below the threshold to observe any improvement in reducing pathophysiological signs of *M. abscessus* infection (Fig. 4). Comparatively, CFTR depletion in zebrafish embryos led to remarkably improved phage responsiveness, recapitulated in improved embryo survival, reduced bacterial burdens and reduced pathophysiological signs such as cords and abscesses (Fig. 5). This is likely due to defective host defences such as reactive oxygen species production, which is known to increase susceptibility to NTM species such as *M. abscessus* and *M. fortuitum* (Bernut et al., 2019; Johansen and Kremer, 2020a), as opposed to being entirely devoid of an immune cell subset, which is essential for infection control. As such, these findings are unsurprising and provide new insight into the requirements for host immunity within the context of phage therapy. Overall, our results suggest that, in animals, phage therapy success not only requires bacterial permissiveness to phage killing but relies also on complex immunophage interactions. This implies the need to explore the patient immunological status as a critical requirement to consider before applying phage therapy, an observation recently emphasised in a patient receiving phage therapy, who rapidly developed a robust humoral response against the therapeutic phages and whereby the phage-neutralising activity correlated with increasing *M. abscessus* bacterial loads (Dedrick et al., 2021). This strongly suggests that the host immune system can represent an important obstacle to phage therapy. Thus, the future of phage therapy will rely on applying optimal treatments adapted to patients and integrating the immune status of each patient into the treatment strategy.

In summary, our easy-to-use *M. abscessus* infection model paves the way for studying the outcomes from the triple partner interactions between the phage, the target bacterial pathogen and host innate immunity. It also supports the development of phage therapies and provides the first framework towards the development of a pre-clinical platform to assess the most successful phage/antibiotic combinations and predictions prior to testing in *M. abscessus* patients.

## MATERIALS AND METHODS

### Mycobacterial strains and culture conditions

*M. abscessus* CIP104536^T^ (S and R variants), *M. massiliense* CIP108297^T^ (S and R variants) and the clinical strains *M. massiliense* GD01 (rough) and *M. abscessus* T56 (rough) were routinely grown and maintained at 37°C in Middlebrook 7H9 broth (BD Difco) supplemented with 10% oleic acid, albumin, dextrose, catalase (OADC; BD Difco) and 0.025% Tyloxapol (Sigma-Aldrich) (7H9^OADC/T^), or on Middlebrook 7H10 supplemented with 10% OADC enrichment (7H10^OADC^). Fluorescent GD01 was generated using the pTEC27 expressing tdTomato (Takaki et al., 2013). Red fluorescent colonies were selected on 7H10^OADC/T^ supplemented with 1000 µg/ml Hygromycin (Euromedex) and maintained in 7H9^OADC/T^ supplemented with 500 µg/ml Hygromycin. Fluorescent *M. abscessus* CIP104536^T^ and *M. massiliense* CIP108297^T^ strains have previously been described (Bernut et al., 2014a). *M. smegmatis* mc^2^155 was grown at 37°C in Middlebrook 7H9 broth as previously described (Jacobs-Sera et al., 2012).

### Drug susceptibility testing

The MICs were determined according to the CLSI guidelines (Woods et al., 2011). The broth micro-dilution method was used with cation-adjusted Mueller-Hinton broth (CaMHB) with an inoculum of 5×10^6^ CFU/ml in exponential growth phase. Each drug dilution was added to the bacterial suspension and incubated for 3-5 days at 30°C. MICs were recorded by visual inspection (Johansen et al., 2020b). Assays were completed in triplicate in three independent experiments.

### Preparation of phage

Phages were grown on *M. smegmatis* mc^2^155 using solid media and recovered by diffusion into phage buffer (68.5 mM NaCl, 10 mM Tris-HCl pH 7.5, 10 mM MgSO_4_, 1 mM CaCl_2_). Phage lysates were filtered through a 0.22 µM filter and used. High-titre cesium chloride-banded phages were prepared and dialysed as previously reported (Dedrick et al., 2019).

### *In vitro* phage-killing assay

Bacteria grown in 7H9 supplemented with 10% OADC and 2 mM CaCl_2_ (7H9^OADC/CaCl^) were harvested in the exponential growth phase and pelleted by centrifugation at 3000 ***g*** for 10 min. To create single-cell suspensions, bacteria were passed through a 26G needle 20 times and sonicated twice for 10 s to separate large aggregates. Bacteria were made to a concentration of 5×10^6^ CFU/ml, and 100 μl bacterial suspension was placed into each well. Mycobacteriophage stock dilutions were added to each corresponding well at the desired MOI (phage:bacteria), mixed well by pipetting and then placed at 37°C for up to 5 days. At the desired time point, bacteria from each corresponding well were 10-fold serially diluted in PBS containing 0.05% Tween 80 to lyse residual extracellular phage, and then plated onto LB agar and placed at 37°C for up to 5 days or until visible colonies emerged.

### Zebrafish maintenance

Zebrafish use was performed in agreement with European Union guidelines for the handling of laboratory animals and was approved by the Direction Sanitaire et Vétérinaire de l'Hérault and Comité d'Ethique pour l'Expérimentation Animale under reference 2020022815234677. All experiments in the current study were performed using the *golden* mutant and macrophage reporter *Tg(mpeg1:mCherry)* lines as previously described (Bernut et al., 2014a, 2015). Zebrafish embryos were obtained and maintained as previously described (Bernut et al., 2015).

### Morpholino injection and *cftr* knockdown

Morpholinos were designed and purchased from GeneTools. A splice-blocking morpholino specifically targeting zebrafish gene *cftr* (ZEBRAFISHIN, ZDB-GENE-050517-20) (5′-GACACATTTTGGACACTCACACCAA-3′) was injected into zebrafish embryos at the one- to four-cell stage (1 mM, 2 nl). Furthermore, details of the morpholino efficacy and specificity and *cftr* knockdown were previously validated as described (Bernut et al., 2019).

### Zebrafish microinjection and infection

At 24 hpf, embryos were dechorionated using Pronase (10 mg/ml; Sigma-Aldrich) for up to 5 min at room temperature, followed by extensive washing in zebrafish water. Following dechorionation, macrophage depletion was achieved by microinjection with either liposomal clodronate or PBS-filled liposomes (Liposoma) (2 nl) via caudal vein injection, as previously described (Bernut et al., 2014a, 2015). At 30 hpf, embryos were anaesthetised in 0.02% tricaine solution and microinjected with fluorescent mycobacteria via caudal vein injection (3 nl containing ≈100 bacteria/nl). Bacterial inoculum was checked *a posteriori* by injection of 3 nl into sterile PBS and plating onto 7H10^OADC^. Following infection, embryos were transferred to 24-well plates (two embryos/well) and incubated at 28.5°C for the duration of the experiment. Embryo age is expressed as dpi.

### Phage treatment in GD01-infected zebrafish

At 24 h post-infection (hpi), embryos were anaesthetised in 0.02% tricaine solution and microinjected with 10-15 nl mycobacteriophage solutions (10^9^ PFU/ml) containing Muddy, Gabriela or a phage cocktail at a final concentration of MOI 50:1 (phage:bacteria) of original bacterial infection inoculum. Control embryos were micro-injected with an identical dilution of phage buffer and PBS, as was performed for the phage-treated embryos. Embryos were microinjected daily between 1 and 5 dpi, with each embryo receiving a total of five phage or PBS injections. Embryos receiving RFB treatment were treated as previously described (Johansen et al., 2020b). Briefly, RFB [stock concentration 10 mg/ml in dimethyl sulfoxide (DMSO)] was diluted to 50 µg/ml in zebrafish water, and embryos were immersed in drug-containing water. RFB was replenished daily between 1 and 5 dpi, with each embryo receiving a total of 5 days of RFB treatment. Control embryos were treated with an identical dilution of DMSO and zebrafish water as was performed for the RFB-treated embryos.

### Zebrafish monitoring and live imaging

Embryo survival was monitored daily based on the presence or absence of a heartbeat. Survival curves were determined by counting dead larvae for up to 12 days, or until uninfected embryos begin to die. At designated key time points post-infection, embryos were anaesthetised in 0.02% tricaine solution and mounted on 3% (w/v) methylcellulose solution for live imaging. Images were taken using a Zeiss Axio Zoom.V16 coupled with an Axiocam 503 monochrome camera (Zeiss). FPC measurements were determined using the ‘Analyze particles’ function in ImageJ. Granulomas were identified based on the co-localisation of fluorescent macrophages and fluorescent GD01. All experiments were completed at least two times independently.

### Statistical analysis

Kill kinetics in the absence or presence of antibiotics were analysed using an unpaired Student's *t*-test. Survival curve analysis was completed using the log-rank (Mantel–Cox) statistical test. Abscess and cord analyses were completed using unpaired Student's *t*-test. Bacterial burden (FPC) analyses were performed using a Kruskal–Wallis one-way ANOVA. All statistical tests were completed using GraphPad Prism (Version 9.0.1).

## Supplementary Material

Supplementary information
