# Supplementary material for: Mycobacteriophage–antibiotic therapy promotes enhanced clearance of drug-resistant Mycobacterium abscessus
Source: Dis Model Mech. 2021 Sep 17;14(9):dmm049159. doi: 10.1242/dmm.049159 (PMC8461822; doi:10.1242/dmm.049159)
Supplement: Supplementary information [file dmm-14-049159-s1.pdf]

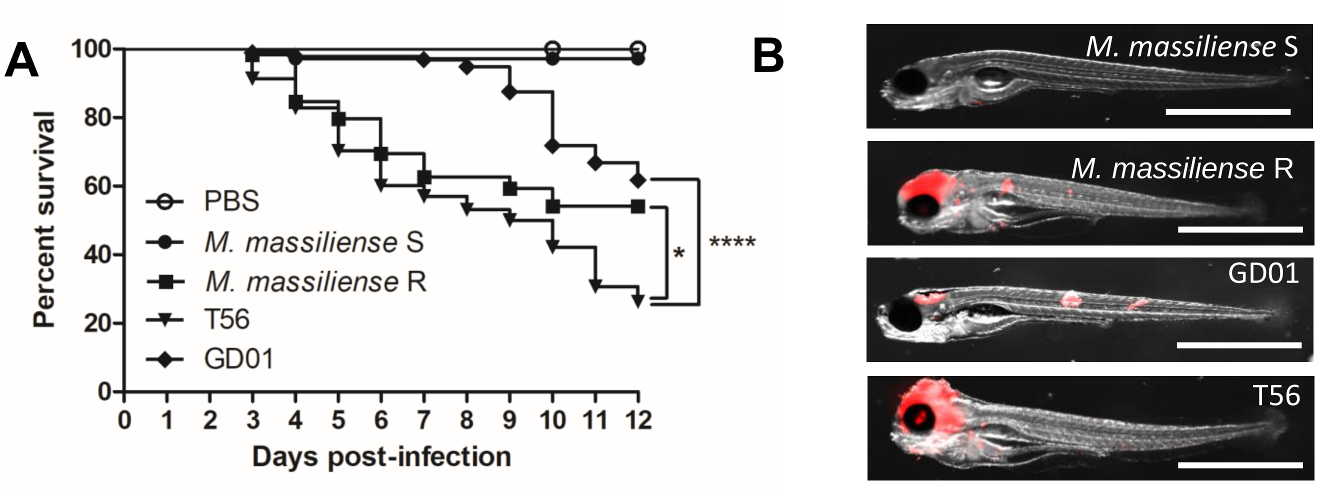

Fig. S1. Comparative virulence of various *M. massiliense* strains in zebrafish embryos. (A) Zebrafish embryos at 30 hpf were infected *via* caudal vein injection with approximately 200-250 CFU of each corresponding strain. Embryonic survival was monitored daily over a 12-day period. Represented data is the merged of 4 independent experiments, with approximately 20 embryos per group. * $P < 0.05$, **** $P < 0.001$. **(B)** Representative images taken at 5 dpi, highlighting the heterogeneity in disease phenotypes between the different isolates. Scale bars represent 1 mm. Red areas highlight the bacterial localisation using fluorescence microscopy. Data shown is the merge of three independent experiments (n=30/group for each replicate).

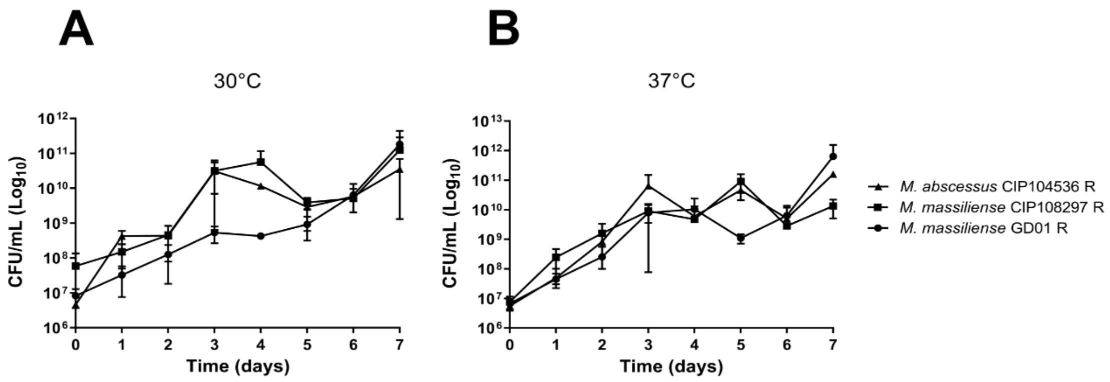

Fig. S2. Growth curves of rough strains carrying pTEC27 at 30°C and 37°C. *M. abscessus* CIP104536^{T}, *M. massiliense* CIP108297 and GD01 were grown for 7 days in Middlebrook 7H9 supplemented with OADC, 0.025% tyloxapol and 500 µg/ml hygromycin. CFU counts determined by plating serial dilutions onto in Middlebrook 7H10 and enumeration of the colonies after 4 days of incubation at 30°C **(A)** or 37°C **(B)**. Data shown is the mean ± standard deviation of two independent experiments run in triplicate.

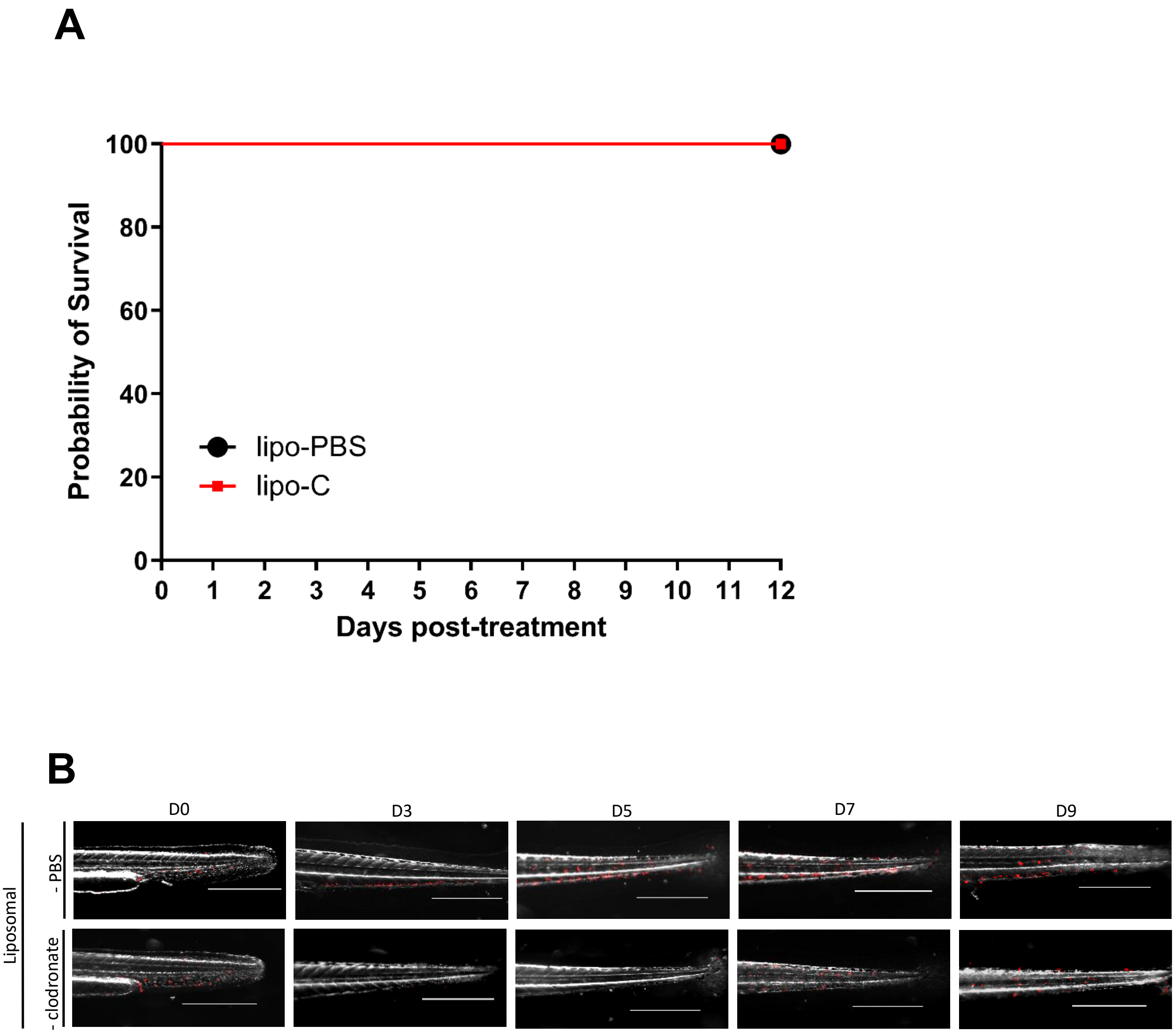

Fig. S3. Impact of liposomal-clodronate on zebrafish survival and macrophage depletion. Tg(*mpeg1:mCherry)* embryos harbouring red fluorescent macrophages were injected with 3 nL of either liposomal-PBS (lipo-PBS) or liposomal-clodronate (lipo-C) in the caudal vein at 24 hpf. **(A)** Embryo survival was monitored daily over a 12 day period (n=30/group). **(B)** Representative images of zebrafish tails showing transient macrophage depletion with lipo-clodronate. Macrophages are labelled in red. Scale bars represent 500 µm.

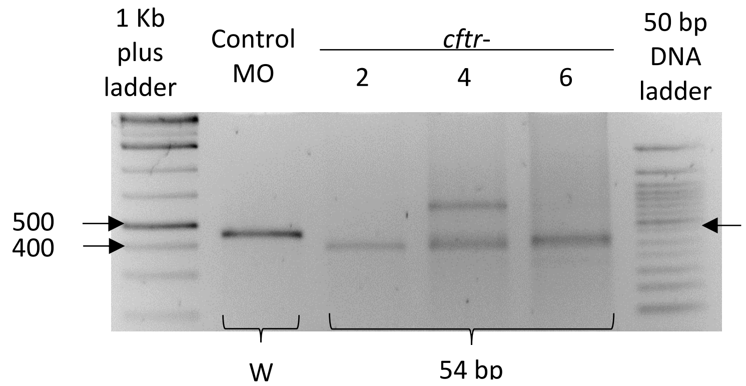

Fig. S4. Targeted knockdown of cftr using splincing morpholino in embryos. *cftr* specific products were amplified from RNA isolated from whole embryos at 2, 4 and 6 dpf. The *cftr*-MO blocks normal splicing resulting a deletion in exon 3, resulting in a 54 bp deletion in exon 3, and leading to the knockdown of CFTR expression (Bernut et al., 2019; Johansen and Kremer, 2020a).

Table S1. Drug susceptibility/resistance profile of *M. massiliense* GD01. Rough *M. abscessus* CIP104536^T and *M. massiliense* CIP108297^T were included for comparison. MIC (µg/mL) were determined after 4 days of incubation, following the CLSI guidelines.

**Strain** (morphotype)	**MIC (µg/mL)**														
	**IPM**	**CFX**	**CLR**[1]	**TGC**	**CFZ**	**AMK**	**CIP**	**ZEO**	**KAN**	**BDQ**	**LNZ**	**GEN**	**RFB**	**Cpd12**	**E JMCh-6**
M. abscessus (R)	16	64	4	1	1	64	128	32	16	0.03	64	64	12.5	0.06	0.125
M. massiliense (R)	32	64	0.5	2	0.5	32	64	>128	16	0.06	64	64	25	0.06	0.125
GD01 (R)	32	64	>128	2	0.5	>128	64	>128	>128	0.015	64	> 128	25-50	0.25	0.125-0.25

IPM, imipenem; CFX, cefoxitin; CLR, clarithromycin; TGC, tigecycline; CFZ, clofazimine; AMK, amikacin; CIP, ciprofloxacin; ZEO, zeocin; KAN, kanamycin; BDQ, bedaquiline; LNZ, linezolid; GEN, gentamycin; RFB, rifabutin; Cpd12, indole-2 carboxamide; EJMCh-6, benzimidazole.

Disease Models & Mechanisms • Supplementary information
